# PTD4 Peptide Increases Neural Viability in an In Vitro Model of Acute Ischemic Stroke

**DOI:** 10.3390/ijms22116086

**Published:** 2021-06-04

**Authors:** Jarosław Mazuryk, Izabela Puchalska, Kamil Koziński, Magdalena J. Ślusarz, Jarosław Ruczyński, Piotr Rekowski, Piotr Rogujski, Rafał Płatek, Marta Barbara Wiśniewska, Arkadiusz Piotrowski, Łukasz Janus, Piotr M. Skowron, Michał Pikuła, Paweł Sachadyn, Sylwia Rodziewicz-Motowidło, Artur Czupryn, Piotr Mucha

**Affiliations:** 1 Laboratory of Neurobiology, Nencki Institute of Experimental Biology PAS, 02-093 Warsaw, Poland; progujski@imdik.pan.pl (P.R.); rafal.platek@pg.edu.pl (R.P.); a.czupryn@nencki.edu.pl (A.C.); 2 Department of Electrode Processes, Institute of Physical Chemistry, Polish Academy of Sciences, Kasprzaka 44/52, 01-224 Warsaw, Poland; 3 Faculty of Chemistry, University of Gdańsk, Wita Stwosza 63, 80-308 Gdańsk, Poland; izabelapuchalska8@gmail.com (I.P.); magdalena.slusarz@ug.edu.pl (M.J.Ś.); jaroslaw.ruczynski@ug.edu.pl (J.R.); piotr.rekowski@ug.edu.pl (P.R.); piotr.skowron@ug.edu.pl (P.M.S.); s.rodziewicz-motowidlo@ug.edu.pl (S.R.-M.); 4 Institute of Biotechnology and Molecular Medicine, 80-172 Gdańsk, Poland; 5 Laboratory of Molecular Neurobiology, Centre of New Technologies, University of Warsaw, 02-097 Warsaw, Poland; k.kozinski@cent.uw.edu.pl (K.K.); m.wisniewska@cent.uw.edu.pl (M.B.W.); 6 NeuroRepair Department, Mossakowski Medical Research Institute PAS, 02-106 Warsaw, Poland; 7 Laboratory for Regenerative Biotechnology, Gdańsk University of Technology, 80-233 Gdańsk, Poland; psach@pg.edu.pl; 8 Department of Biology and Pharmaceutical Botany, Faculty of Pharmacy, Medical University of Gdańsk, 80-416 Gdańsk, Poland; arpiotr@gumed.edu.pl; 9 MedVentures Company, 60-141 Poznań, Poland; j.medventures@gmail.com; 10 Laboratory of Tissue Engineering and Regenerative Medicine, Department of Embryology, Medical University of Gdańsk, 80-210 Gdańsk, Poland; pikula@gumed.edu.pl

**Keywords:** arginine-rich peptides, cell-penetrating peptides, excitotoxicity, ischemic stroke, neural viability, neuroprotection, neurotoxicity, peptide conformation, PTD4, Tat(49–57)-NH_2_

## Abstract

Ischemic stroke is a disturbance in cerebral blood flow caused by brain tissue ischemia and hypoxia. We optimized a multifactorial in vitro model of acute ischemic stroke using rat primary neural cultures. This model was exploited to investigate the pro-viable activity of cell-penetrating peptides: arginine-rich Tat(49–57)-NH_2_ (R^49^KKRRQRRR^57^-amide) and its less basic analogue, PTD4 (Y^47^ARAAARQARA^57^-amide). Our model included glucose deprivation, oxidative stress, lactic acidosis, and excitotoxicity. Neurotoxicity of these peptides was excluded below a concentration of 50 μm, and PTD4-induced pro-survival was more pronounced. Circular dichroism spectroscopy and molecular dynamics (MD) calculations proved potential contribution of the peptide conformational properties to neuroprotection: in MD, Tat(49–57)-NH_2_ adopted a random coil and polyproline type II helical structure, whereas PTD4 adopted a helical structure. In an aqueous environment, the peptides mostly adopted a random coil conformation (PTD4) or a polyproline type II helical (Tat(49–57)-NH_2_) structure. In 30% TFE, PTD4 showed a tendency to adopt a helical structure. Overall, the pro-viable activity of PTD4 was not correlated with the arginine content but rather with the peptide’s ability to adopt a helical structure in the membrane-mimicking environment, which enhances its cell membrane permeability. PTD4 may act as a leader sequence in novel drugs for the treatment of acute ischemic stroke.

## 1. Introduction

Stroke is characterized as an acute pathological decrease in blood flow in the brain, which lasts longer than 24 h and often leads to neurological deficits or death. Acute ischemic stroke (AIS) accounts for 87% of stroke cases and is a leading cause of disability and death worldwide [1,2]. In 2017, stroke-related health care cost 32 European countries around €60 billion [3]. At the cellular level, the complex pathophysiology of AIS mainly involves excitotoxicity and inflammation [4,5]. In detail, the excitotoxicity follows primary events that occur upon the disruption of the blood–brain barrier, i.e., nutrient deprivation and acute hypoxia. These malfunctions are also associated with uncontrolled depolarization of membrane potential and mitochondrial dysregulation, causing cellular respiration impediment and deviated aerobic glycolysis, and leading to lactic acidosis. The ultimate outcome of this morbid cascade is an irreversible loss of neuronal function along with neuronal death.

Minimization or reversion of deleterious AIS impacts on neurons, ascribed to post-ischemic neuroprotection, is a pharmacological challenge. Currently, the only available AIS treatment strategy is re-establishing perfusion through the obstructed blood vessels with pharmacologic or mechanical thrombolysis. Thus far, intravenous administration of recombinant serine protease tissue-type plasminogen activator (rtPA), i.e., the only FDA-approved drug for AIS, has its intrinsic limitations such as a short therapeutic time window (about 4.5 h from the incident of stroke) and the unavoidable risk of subsequent hemorrhage [6,7].

One of the most recent approaches to reduce AIS-related neuronal death is the application of cell-penetrating peptides (CPPs) as neuroprotective agents. CPPs have emerged as a powerful medicine tool owing to their exceptional ability to cross cell membranes [8,9]. The era of CPPs in biomedicine originated with the discovery of the *trans*-activator of transcription HIV-1 Tat protein [10,11]. The unusual activity of the CPPs is endowed by the Tat transduction domain (PTD), the arginine-rich motif (ARM, amino acids 49–57), a uniquely evolved retroviral protein module [12]. The PTD is a blood–brain barrier (BBB) shuttle peptide with an intrinsic membrane crossing activity that facilitates the cargo translocation across an intact BBB [13,14]. Moreover, the PTD-mediated translocation through the cell membrane has been shown to depend on the arginine content of the PTD and other arginine-rich peptides (RRPs), i.e., a group of peptides possessing size from 4 to 40 amino acids, positive net charge (+2 to +20), one or more positively charged arginine residues that comprise from 20 to 100% of the peptide, as well as other positively charged amino acids [15,16]. Sequence modifying studies demonstrated that cell membrane translocation of PTD4 peptide, which has a lower number of arginine residues than Tat(49–57) [17,18], was accomplished more preferably than in the case of the latter [19,20]. (Table 1).

This remarkable feature of the RPPs was thrivingly encompassed in the cellular delivery of neuroprotective cargoes [21]. Along with the pronounced arginine-content influence on the cellular permeability potency of the RRPs, it strongly correlates with neuroprotective activity. In a series of studies, it was shown that exceeding the optimal arginine residues number may attenuate pro-viable activity [5,22,23]. Moreover, neurotoxicity of specific Tat peptides and full-length native HIV-1 Tat protein was also reported as approximately 15–50% of HIV-infected patients suffer from HIV-associated neurocognitive disorders (HAND), dementia, mild or asymptomatic neurocognitive impairment, and encephalitis [24,25]. Because the neurotoxicity mechanism of Tat peptides differs significantly from that of HIV-1 Tat protein, comprehensive studies of Tat peptides’ impact on neural cells appears as a matter of paramount importance.

Tat(49–57)-NH_2_ is commonly used in neuroscience as a selective cell-penetrating transporter [21,26,27]. Classic studies demonstrated that Tat peptides’ neuroprotection strongly depends on their electrostatic and conformational properties [5,16,28]. More recently, however, the idea that the ability of a peptide to exert neuroprotection depends on its potential to penetrate the neuronal membrane began to prevail [29]. Although several parameters are essential for this internalization mechanism, recent focuses suggest that conformational interactions with membrane phospholipids play a crucial role during cellular uptake [30].

The therapeutic potential of RRPs for reversing AIS symptoms has emerged from the screening of neuroprotective drugs capable of the immediate preservation of neurons in the ischemia-stricken brain. Thus far, vast and convincing data demonstrate this propensity for RRPs. Moreover, these pro-viability merits were matched with the net charge of the RRPs [5,22,28]. It was clearly proved that the high cationic charge enables electrostatic binding of the arginine guanidinium groups with heparan sulphate proteoglycans, sialic acid residues on glycosphingolipids, or phosphate head groups present in membrane phospholipids [5,31]. Furthermore, this capacity’s effectiveness enhances with increasing peptide length, the number of arginine residue, and net charge, wherewith 15 arginine residues provide maximal efficacy [5,22,23]. In physiological conditions, this mechanism not only impacts the neuronal endocytic progression and endosomal peptide transduction but also stimulates the internalization of cell surface ion channels and receptors. This, in turn, decreases intracellular calcium ion influx and the risk of fatal excitotoxicity [31].

Various in vitro models of AIS have been established in the recent decade [32]. Most common platforms refer to brain microvascular endothelial cells [33], implemented with astrocytes and pericytes [34], extracellular matrix components [35], or associated with circulating immune cells [36]. Other models involve primary neuronal-astrocytic cultures [37], embryonic stem cells [38], induced pluripotent stem cells [39], and human conditionally immortalized neural stem cells [40].Future studies shall focus on 3D co-cultures seeded on hydrogel scaffolds and biomatrices [41] or cultivated in microfluidic systems [42]. These sophisticated models enable physiologically relevant and multi-level in vitro studies on energy depletion, excitotoxicity, inflammation, and drug delivery to AIS [32]. On the other hand, due to complexed structures, ethical issues, and expensive and labor-consuming fabrication, these models are hardly useful for high-throughput drug screening, drug-cell interaction studies, and pre-clinical trials [32,43].

For this motif, in the present study, we used a simple model of primary neural cells to investigate the relationship between basicity, secondary structure, and pro-viable activity of Tat peptides. We then used a multifactorial in vitro model of AIS and a series of Tat analogues to test which of these parameters affects neuroprotection of the peptides. We demonstrated that the PTD4 peptide displays higher protective activity than the Tat(49–57)-NH_2_ peptide, suggesting that conformational features of these peptides and their ability to translocate through the cell membrane, rather than the arginine content and a positive net charge, indicate their ameliorating capability of neural viability preservation.

## 2. Results

### 2.1. Ala-Substitution Favors Helicity of PTD4 Compared to Tat(49–57)-NH_2_

**Figure 1:**
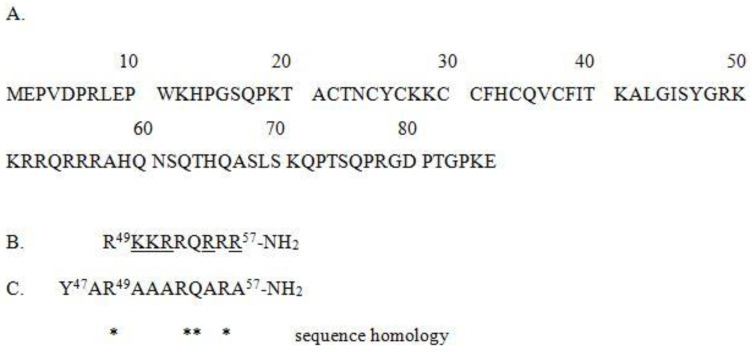
(**A**) Amino acid sequences of the “canonical” variant of HIV-1 Tat (P04608-1 identifier of the UniProt database), (**B**) Tat(49–57)-NH_2_ with underlined Ala-substituted residues, and (**C**) PTD4. Asterisks (*) indicate positions of sequence homology (R, R, Q, R).

In general, arginine-rich peptides such as Tat(49–57) cannot adopt a helical structure [44]. In aqueous solutions, the polyarginine peptides form unstructured [45] or polyproline type II helical conformations [46,47,48]. Most likely, this phenomenon results from a strong repulsive interaction between the quinidine groups of the arginine residues. As already demonstrated, modeling the Tat’s ARM structure in order to obtain the most stable helical conformation has led to the design of the PTD4 peptide (see Figure 1), in which the replacement of particular arginine residues by alanine residues resulted in a Tat(49–57)-NH_2_ analogue with enhanced membrane transduction potential [45].

**Figure 2:**
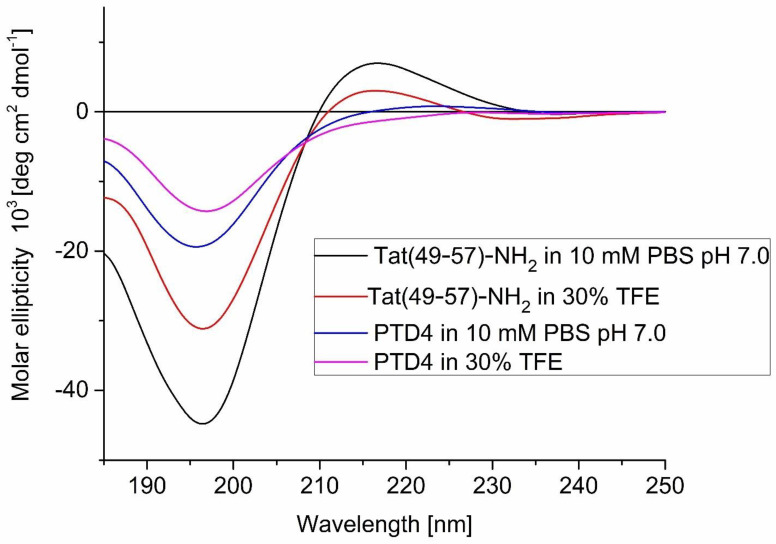
CD spectra of Tat(49–57)-NH_2_ and PTD4 peptides in 10 mM PBS, pH 7.0 and 30% (*v*/*v*) TFE.

In order to verify how conformational structures of the peptides impact their potential to cross the cell membrane, we inquired whether a replacement of Arg with Ala amino acid residues increases the peptides’ helicity. The influence of this Ala-substitution on the conformational properties of the Tat(49–57)-NH_2_ and PTD4 peptides was evaluated by CD spectroscopy. The CD spectra of Tat(49–57)-NH_2_ aqueous solutions revealed distinct polyproline type II conformations (Figure 2). The CD spectrum presented a strong negative minimum at λ ≈ 195 nm and an additional maximum at λ ≈ 215 nm. In contrast, the CD spectra of PTD4 possessed a weak minimum at λ ≈ 195 nm with an additional wide maximum at λ ≈ 220 nm, characterized by lower intensity and increased width compared to those of Tat(49–57)-NH_2_. This indicated a more unstructured (random coil) conformation of PTD4 compared to Tat(49–57)-NH_2_. Together, these outcomes presented the effect of the Ala-substitution procedure on the conformational properties of Tat(49–57)-NH_2_.

### 2.2. Conformational Structure of PTD4 Predisposes It to Enter the Cell Interior

Since the peptide conformation-based interactions with the cell membrane or membrane receptors may contribute to CPP pro-viable properties, we compared conformational behaviors of Tat(49–57)-NH_2_ and PTD4 peptides in 30% (*v*/*v*) TFE solution, which is an excellent alpha-helix inducer and, as such, may mimic the hydrophobic interior of the cell membrane [49,50]. In this condition, peptides tend to adopt a stable secondary, mainly helical, conformation that predisposes them for natural conformational behavior [17,18,51,52]. TFE-induced folding processes in the peptide structures were analyzed by CD spectroscopy. The CD spectra of Tat(49–57)-NH_2_ and PTD4 peptides displayed negative maxima at λ ≈ 195 nm. However, a significant difference in intensity of molar ellipticity was observed compared to spectra acquired in aqueous solutions. In the case of Tat(49–57)-NH_2_, a significant intensity decrease of a positive maximum at λ ≈ 215 nm was also observed. Regarding PTD4, the shallow maximum at λ ≈ 225 nm was no longer visible and was replaced by a shallow, weakly scratched minimum at λ ≈ 225 nm. In contrast, Tat(49–57)-NH_2_ exhibited a shallow positive maximum at this wavelength. The CD spectra recorded in 30% TFE showed evident conformational changes compared to those recorded in an aqueous solution. This indicated that for Tat(49–57)-NH_2_ the content of type II polyproline helix structure decreased and the random-coil content increased, whereas, for the PTD4 peptide, a small minimum at λ ≈ 225 nm indicated the formation of an α-helical conformation. These structural differences predispose PTD4 to penetrate the cell interior more effectively than Tat(49–57)-NH_2_.

### 2.3. PTD4 Adopts the Favorable Structure in the Membrane-Mimicking Environment

To confirm structural differences between PTD4 and Tat(49–57)-NH_2_, we calculated molecular dynamics (MD) with no constraints for both peptides in water and 30% TFE. Afterwards, in the 100 ns-long dynamics simulations in the AMBER full-atomic force field, the structures were divided into 10 clusters (see Figure S1a,b for water and Figure S1c,d for 30% TFE, respectively; see Supplementary Materials). The percentage of molecules in each cluster is presented in Figure S1. As observed, cluster 1 (cluster with the largest conformation population) and cluster 2 (cluster with the second-largest conformation population, see Figure S1) occurred as the most abundant and summed up to ~60% of all conformations.

**Figure 3:**
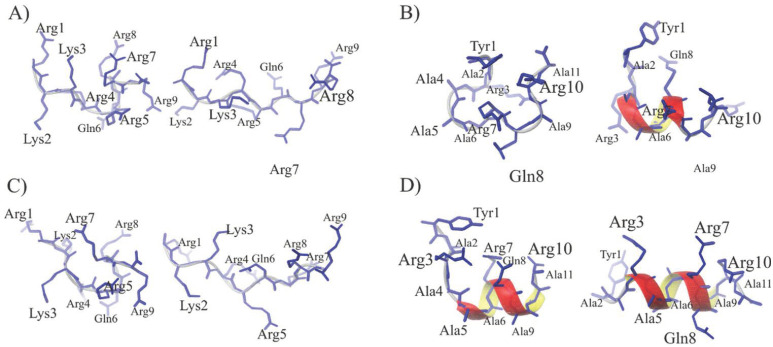
Spatial structures of the two most dominant clusters calculated for Tat(49–57)-NH_2_ in water (**A**) and 30% TFE (**C**), and for PTD4 in water (**B**) and 30% TFE (**D**). On the left, cluster 1; on the right, cluster 2.

Representative structures obtained for these dominant clusters are presented in Figure S1. In both solvents, Tat(49–57)-NH_2_ formed mainly a disordered conformation (cluster 1) and classic type II polyproline helix (cluster 2). As shown in Figure 3A,C, the positively charged amino acid residues extruded out the Tat(49–57)-NH_2_ peptide due to strongly repulsive positive charges present on the side chains. As a result, the Arg and Lys residues were mostly located on the other two sides of the type II polyproline helix. Hence, this adopted spatial disposition was suitable for interaction with the cell membrane in the first step of peptide internalization [53,54]. In contrast, the PTD4 peptide also displayed a disordered conformation (cluster 1) but with a slight tendency to form the helical structure in the Ala4-Gln8 region (cluster 2). In 30% TFE though, the PTD4 peptide exhibited a more pronounced tendency towards the helical structure formation. In this condition, in cluster 1 the helix was formed in the Ala5–Arg10 region, whereas in cluster 2 the formation of the helix was in the Arg3–Ala9 region. These analyses demonstrated that in the hydrophobic, membrane-mimicking environment, such as 30% TFE solution, PTD4 adopted the helical conformation (Figure 3D), characterized by an arginine residue stack present on the same side of the helix. Since a helical conformation and an increased arginine content in the peptide enhance its translocation through the cell membrane, it is expected that PTD4 will effectively interact with the neuronal membrane. Future investigations using other membrane mimetics, such as sodium dodecyl sulphate, and small or large unilamellar vesicles, or lipid bilayers, shall reveal details of the PTD4 secondary structure and its behavior upon contact with the cell membrane.

### 2.4. PTD4 Expresses no or Minor Neurotoxicity in Primary Neural Cortical Cultures

**Figure 4:**
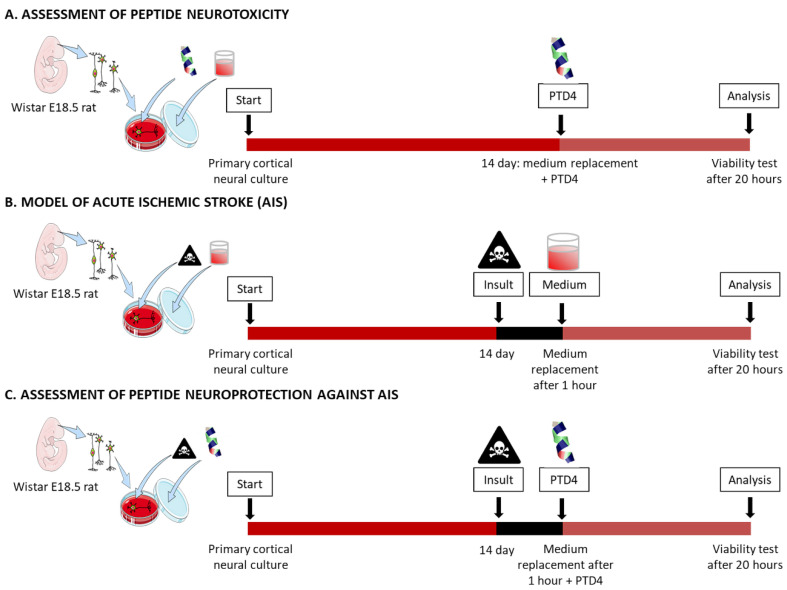
Experimental designs: peptide neurotoxicity (**A**), in vitro model of AIS (**B**), and peptide neuroprotection against AIS (**C**).

**Figure 5:**
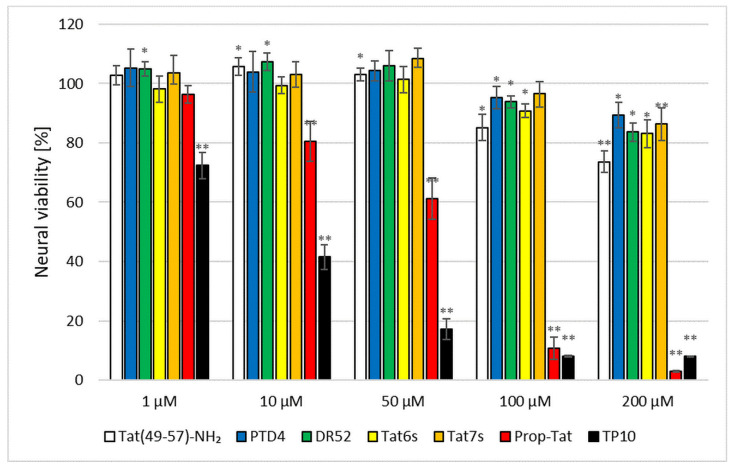
Peptide neurotoxicity in the cortical culture. Non-insulted cells were treated as 100% control. Data demonstrate relative measurements to control ± SD. Two-way ANOVA statistics: *n* = 3; * *p* < 0.05, ** *p* < 0.001.

**Figure 6:**
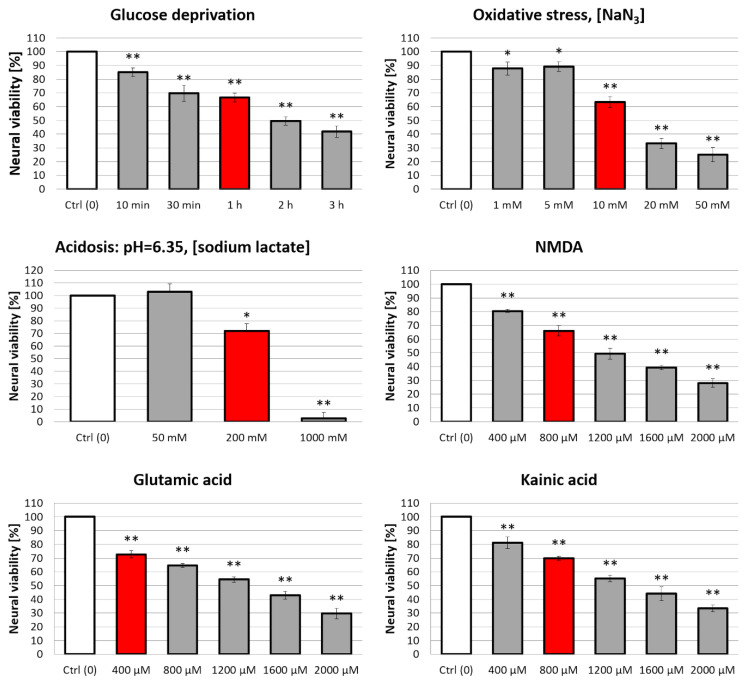
Optimization of an in vitro model of AIS. Red bars indicate conditions causing a 30–40% decrease in neural viability. These conditions were then selected for assessments of the neuroprotective activity of tested peptides. Non-insulted cells were treated as 100% control. Two-way ANOVA statistics: *n* = 3; * *p* < 0.05, ** *p* < 0.001. Each statistical significance asterisk refers to a respective control (white bar) ± SD.

Being a CPP/RRP group member, Tat(49–57)-NH_2_ expresses ambiguous activity in terms of its influence on neuronal metabolism and signal trafficking within the brain [23]. Hence, pharmacological application of Tat(49–57)-NH_2_, either as a drug delivery system or a stand-alone neuroprotective agent, would require strict control in both dose-dependent and spatio-temporal manners. Despite the well-documented inherent neuroprotection of RRPs, special attention must be paid to their neurotoxic activities. What is more, *C*-terminal amidation may increase the neuroprotective effect of the peptide, whereas, conversely, *N*-terminal acetylation generally decreases it, as was shown for Arg9 peptide [55]. The neurotoxic influence of these post-translational modifications (PTMs) was proven to be dose-dependent and, basically, to enhance peptide stability against enzymatic proteolysis in physiological conditions [56]. Similarly, an ameliorating effect was observed upon the position-specific D-stereoisomerization of CPPs [57]. Comprehensive studies have demonstrated that the D-amino acid-bearing CPPs display higher protease resistance [58] and undergo facilitated cell type-dependent internalization [59]. The effectiveness of the cell entry has been reported to depend upon the number of D-arginine residues in CPPs [60]. Finally, alkylation of CPPs has also been demonstrated to enhance their performance [44]. Such modification of CPPs may change its translocation through the cell membrane or the specificity of membrane receptor recognition [61,62]. These, in turn, may cause peptide neurotoxicity or neuroprotection. To verify these aspects with regard to Tat RRPs, a series of Tat analogues were synthesized (Table 1), and their neurotoxicity was assessed in primary neural cultures (Figure 4, Figure 5 and Figure 6). Apart from Tat(49–57)-NH_2_ and PTD4, the following modifications were analyzed: *N*-acetylation (Tat6), arginine methylation (Tat7), *N*- propiolylation (Prop-Tat), and D-Arg substitution (DR52). Besides, we analyzed TP10 peptide, a commonly used CPP [9], that does not bear amino-acid sequence homology to Tat(49–57)-NH_2_. This set of analogues contains typical PTMs that diversify these peptides with regard to the net charge and hydrophobicity, and conceivably, with regard to neuroactivity. Prop-Tat analogue was included in this series to characterize the neurotoxicity of the alkyne-bearing component of click-chemistry reaction.

Neural cultures were exposed to each peptide for 20 h, after which time their viability was assessed using a conventional spectrofluorimetric CellTiter-Blue^®^ (CTB) assay (Figure 4) [63].The CTB assay was selected to replace a standard MTT agent. Although MTT was shown to perform excellent effectiveness in stable cell lines [64], several reports demonstrated its ill-disposed accuracy in the assessment of drug-dose response in cell lines [65,66] and neuron-astroglia cultures [67]. Figure 5 presents results of neurotoxicity. At low concentrations (up to 50 µM), the Tat peptides demonstrated no or low neurotoxicity. In this concentration range, PTD4 neurotoxicity was negligible, albeit significant neurotoxic effects of Tat peptides were visible at higher concentrations (>100 µM), at which the most toxic impact was observed for Tat(49–57)-NH_2_. Within this concentration range, the Tat analogues displayed slightly lower toxicity than that of Tat(49–57)-NH_2_. In contrast, acute neurotoxicity was evident in the cases of Prop-Tat and the TP10 peptide. The neurotoxic impact of these agents was visible in a concentration of 1 µM, TP10 being the most pronounced. In high concentrations (>100 µM), Prop-Tat and TP10 peptides reduced neural viability to <10%. Among all the peptides tested, TP10 displayed the highest toxicity in a wide concentration range. In summary, the Tat peptides studied showed no or negligible neurotoxicity in a concentration below 100 μM. The highest nontoxic peptide concentration of 50 μM was used to assess their pro-viable effects. Because PTD4 displayed the lowest neurotoxicity among Tat analogues used in this study, this peptide was evaluated in terms of increasing neural viability in an in vitro model of AIS.

### 2.5. PTD4 Is Pro-Viable in an In Vitro Model of Acute Ischemic Stroke

We selected Tat(49–57)-NH_2_ and PTD4 peptides to validate the influence of Arg^52,55,57^, Lys^50,51^→Ala substitutions, that resulted in the peptide net charge decrease (from +9 to +4, respectively) in the neural survival induced by these peptides (Table 1). This influence was evaluated in an optimized multifactorial cellular model of AIS. The optimization of this model is shown in Figure 6. The conditions that caused a 30–40% decrease in the neural viability within one hour of the exposure period (red bars) were selected for further assessment of pro-viable activities of the peptides.

In this model, rat primary neural culture was individually pre-treated with a series of AIS-related neurochemical injury factors (insults) such as glucose deprivation, oxidative stress (OS, induced by sodium azide), lactic acidosis, and excitotoxicity (induced separately by NMDA, glutamic acid or kainic acid). It is to be noted that, sodium azide, a typical inhibitor of complex IV in the respiratory chain, has been commonly used to induce OS in vitro and in vivo [68,69,70,71]. Likewise, acidosis was optimized using sodium lactate. Lactate is a metabolite of anaerobic respiration that occurs upon energetic deprivation, and has been widely used to mimic acidosis in excitotoxicity research [72,73,74]. The model was optimized based on classical studies. Excitotoxic factors: glutamic acid, kainic acid, and NMDA were applied as described elsewhere [16,22,28,31,55,75]. The results of this optimization are presented in Figure 6. The intensity of these insult conditions was optimized in order to reduce the neural viability to the level of only 60–70% of the initial value (control conditions). These cells that were initially pre-exposed to normalized insult factors were subsequently exposed to Tat(49–57)-NH_2_ or PTD4 in the concentration of 50 µM, which was selected according to the neurotoxicity assessment (Figure 5).

**Figure 7:**
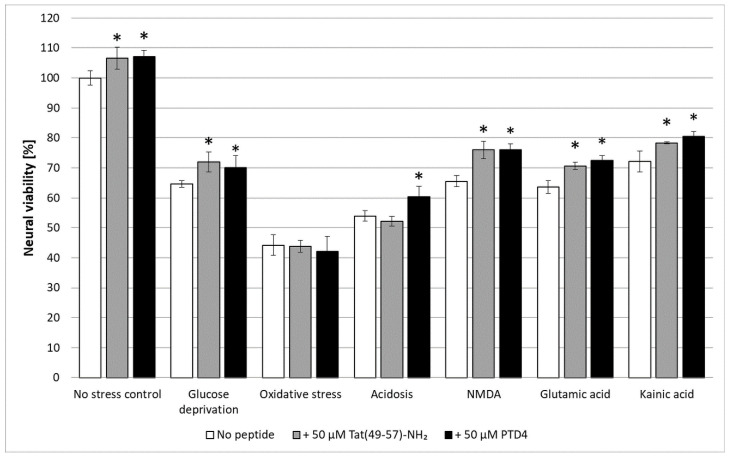
Pro-viable activity of Tat(49–57)-NH_2_ and PTD4 peptides in a multifactorial in vitro model of AIS. Non-insulted cells were treated as 100% control. Two-way ANOVA statistics: *n* = 3; * *p* < 0.05. Each statistical significance asterisk refers to a respective no-peptide control (white bar) ± SD.

The PTD4 peptide was chosen for this evaluation because of its best performance in neural viability assessment among all Tat tested analogues. The highest non-toxic concentration was selected, however, using a wider range of concentration (1–10 µM) that would give a more conclusive insight into the peptide’s activity [76,77]. The results of neural viability validation are presented in Figure 7. A post-insult 20-h exposure to each peptide revealed their significant survival-stimulating properties against almost all insults except sodium azide-induced oxidative stress. The yields of this ameliorating effect were similar for both peptides, amounting to about 10%. The most pronounced peptide activity was observed as alleviation of energetic deprivation and excitotoxicity. The peptides did not prevent neural death caused by cellular respiration inhibition. In the case of acidosis, only PTD4 displayed pro-viable activity.

## 3. Discussion

According to WHO reports, strokes are the second leading cause of death and the third leading cause of disability worldwide. This vast group of cerebrovascular disorders is commonly divided into ischemic and hemorrhagic strokes. Whilst successful hemorrhage prevention usually depends on instant surgical bloodstream stemming, the physiological restoration of blood flow within impaired tissue in ischemic penumbra is possible by pharmacological treatment immediately after the insult [78,79,80]. Recently developed, brain-targeted drug-delivery systems employ CPPs, peptides with an intrinsic ability to effectively traverse the cell membrane and an inherent capacity for neuroprotection [81]. Despite the identification of over a thousand CPPs, recent clinical trials have mostly concerned the neuro-application of Tat peptides, i.e., fragments or analogues of the ARM of HIV-1 Tat protein, that display beneficial neuro-oriented propensities, such as structural flexibility, modification easiness, receptor binding selectivity, and crossing the blood–brain barrier (BBB) [13,82]. All of these taken together make CPPs the perfect candidates for the starting point for the future pharmacological therapy of AIS.

Clinical application of Tat peptides as a cure for neurological diseases is troublesome due to ambivalent outcomes of their activity. Despite the undeniably deleterious neuropathology of HIV-1 Tat protein, high-throughput in in vitro and in vivo studies demonstrated dose-dependent anti-stroke neuroprotection of HIV-1 Tat-derived Tat peptides and their RRP analogues [22,83,84]. The aim of our study was to verify whether conformational properties increased pro-viable activity of arginine-rich Tat(49–57)-NH_2_ and its less basic analogue PTD4. First, we analyzed the neurotoxicity of selected Tat analogues in the 1–200 µM concentration range. In general, the native sequence of Tat(49–57)-NH_2_ and its analogues did not show significant differences in neurotoxicity up to a concentration of 50 µM. Above this value, a progressive increase in neurotoxicity of the peptides was visible. At higher concentrations, the Tat analogues were less neurotoxic than the native sequence, thus indicating that PTMs may modulate and inhibit the neurotoxic effect. For Prop-Tat and TP10, acute neurotoxicity was observed even at low peptide concentration, which may be associated with their high ability to translocate through the cell membrane or bind to a specific membrane receptor. These results show that not all CPPs are useful as potential neuroprotective agents and suggest that, in some cases, the alkynyl components of the click-reaction may be neurotoxic. Future elucidation using the structure–activity relationship (SAR) method will shed more light on critical molecular properties of PTM Tat analogues required for their neuroprotection.

Potential neuroprotective activity of PTD4, in the highest non-toxic concentration (50 µM), was analyzed in an in vitro model of AIS. Previous studies showed the use of sodium azide and sodium lactate to induce oxidative stress and acidosis, respectively [68,69,70,71,72,73,74]. As well, a series of studies was executed to optimize oxygen-glucose deprivation and excitotoxicity [16,22,28,31,55,75]. PTD4 exhibited lower toxicity and mild but higher neuroprotection in comparison to the native sequence of Tat(49–57)-NH_2_. In particular, both peptides were significantly neurotoxic at concentrations above 100 μM, but this effect for Tat(49–57)-NH_2_ was markedly higher. The possible reasons for the neurotoxicity of both peptides may be correlated with already-established Tat-induced elevation of cytosolic calcium levels [85], excitotoxicity [86,87], mitochondrial dysfunction and oxidative stress [88], and blocked autophagy [89], as well as alterations of signal trafficking [24,89]. From this perspective, the PTD4 peptide emerges as a better-performing anti-AIS drug candidate than Tat(49–57)-NH_2_.

The maximal active concentration predisposing PTD4 and Tat(49–57)-NH_2_ peptides for neuronal targeting was as high as 50 µM. Previous studies, however, demonstrated an augmenting effect of lower doses (0.1–5 µM), yet added prior to the insult and for a shorter time (from 15 min to 2 h) [22,28,31,83,90]. This discrepancy may result from different conditions and doses of insults applied in these studies. In our study, we applied an increased concentration of the insult to provide mild, but sufficient neural mortality (~40%). Therefore, the peptide concentration and exposition duration to the peptides were increased accordingly [75,91,92]. In our model, peptide treatment modestly inhibited neural death caused by all insults, excluding sodium azide-induced oxidative stress. The effects of PTD4 and Tat(49–57)-NH_2_ differed in the case of acidosis as the former exhibits ameliorating activity. Despite well-documented data about the high vulnerability of neurons to acidic pH and the destructive impact of lactic acid [74,93,94,95], it is not clear whether CPPs may ameliorate the effects of acidosis. To add, CPPs display pronounced protection against mitochondrial dysfunction [96,97,98]. Our results shed new light on this activity of several RRPs in lactic acidosis.

Comparing the native Tat(49–57)-NH_2_ and PTD4, the latter appeared as a better-performing neuroprotectant in the AIS model. Mechanistically, this may indicate a possible correlation between neuroprotection and the structural and acid-base peptide properties. Seemingly, pro-viable activity of the peptides is more likely enhanced by facilitated intracellular peptide uptake than the net positive charge of a peptide. This observation is consistent with previous reports, which showed that the efficiency of peptide translocation across the cell membrane is almost thirty times higher in the case of PTD4 compared to Tat(49–57)-NH_2_ [99,100]. Specific properties of Tat(49–57)-NH_2_ and PTD4, such as relatively similar basicity but different net charges (Table 1), may imply diverse mechanisms of trans-membrane internalization of these peptides. The results presented in our study showed that even though the unstructured folding in both peptides was observed in physiological conditions, a helical conformation is adopted only by the PTD4 peptide.

Stroke-related Tat-induced neuroprotection has been demonstrated and exploited in pre-clinical studies and a recently concluded Phase III clinical trial on nerinetide, an eicosapeptide neuroprotectant that interferes with PSD-95 in human ischemia-reperfusion and occurs with rapid endovascular thrombectomy in AIS patients (ClinicalTrials.gov Identifier: NCT02930018, [101]). Yet, unraveling the molecular mechanism underlying its activity remains a challenging task. As is known, Tat(48–57) neuroprotection in stroke models strongly depends on the route of administration of the peptide, e.g., in contrast to intraperitoneal delivery, the intracerebroventricular injection of this peptide enabled neuroprotection against cerebral ischemia [83,102]. Besides, fused RRPs were used for symptomatic therapies of global cerebral ischemia [103] and focal ischemic stroke (PTD fused with BCL-XL [104], GDNF [105], and HSP70 [106]). In this context, our results show that the direct delivery of Tat(49–57)-NH_2_ on pre-insulted neural cells provides neuroprotection against several neurochemical impacts of stroke. We also showed similar neuroprotective properties of PTD4, which was already demonstrated to reverse stroke impacts when administrated intravenously [13,14]. This intrinsic in vitro neuroprotection of PTD4 against stroke is in agreement with data already obtained for other RRPs [104,107,108,109]. Yet, in contrast to these studies, which correlate the RRP neuroprotection with the electrochemical properties and arginine content, the CD spectra presented in our study, indicate a tendency of PTD4 to adopt a helical conformation in a membrane-mimicking environment. This structural feature of PTD4, in addition to the lower basicity than that of Tat(49–57)-NH_2_, suggests that the traversing capability of PTD4 may originate from the helicity of this peptide rather than from its net charge.

As a BBB shuttle peptide, PTD4 is expected to interact with the cell membrane to enter brain parenchyma [13,14], either in a receptor-independent fashion [110] or via endocytosis or adsorptive-mediated transcytosis [111]. On the contrary to the endocytosis of regular peptides, during which these peptides are encapsulated in the endocytic vesicle, PTD4, as well as other RRPs, enter the cell interior together with an outer membrane receptor or transmembrane channel, which removes them from the outer cell surface [20,112,113]. As known, RRP reduce an excitotoxic calcium influx to the cell interior by their interaction with glutamate agonist receptors [22,112,114]. This interaction induces endocytic internalization of calcium ion channels and transporters, such as an NR2B subunit of NMDA receptor (NMDAR), i.e., a pivotal modulator of excitotoxic neuronal calcium influx and the damaging of signaling in neurons [31]. However, the experimental data on this topic is still ambivalent [115] as Tat peptides were demonstrated either to inhibit NMDAR and lower the toxic intracellular calcium ions level [5,55], or else to trigger glutamate- or NMDA-induced calcium influx [115,116]. From this perspective, PTD4 and Tat(49–57)-NH_2_, assessed in our in vitro model of AIS, displayed significant neuroprotection against excitotoxicity. Similar ameliorating effects were obtained for hybrid Tat peptides, which were shown to inhibit the activity of the kainite receptor and NMDA-NR2B subunit [108], as well as of NMDAR–PSD-95 complex formation in the intracellular part of NMDAR [31], and which may induce blocking nitric oxide synthase (NOS) activation and nitric oxide (NO) production in mitochondria [31].

Additionally, because mitochondria-penetrating RRPs are capable of indirect prevention of ATP synthesis, maintenance of cytochrome *c* integrity and attenuation of calcium influx-induced neurotoxicity [31], it may be speculated that Tat(49–57)-NH_2_ and PTD4 exhibit an indirect protecting effect against pathological processes occurring in mitochondria, such as glucose deprivation, as indicated by outcomes of our study. The already described mechanisms of Tat and other RRPs-induced neuroprotection mention the stabilization of mitochondrial membrane as well as the inhibition of furin, i.e., a calcium-dependent protein convertase [117]. These pro-viable activities forestall the maturation of proteins involved in stroke-induced death signaling pathways in neurons. Besides, the participation of RRPs in the downregulation of voltage-gated CaV2.2, CaV3.3, and NCX3 and TRPV1 ion channels was reported as well [31].

In summary, our results may be the first inceptive for the discovery of the mechanistic details of Tat(49–57)-NH_2_ and PTD4 peptide. Aside from their well-established activity as drug delivery systems in anti-AIS therapy, these peptides may display inherent neuroprotection against AIS. This pro-viable activity in neural cells may be correlated with capability of these peptides to adopt a helical conformation rather than with the arginine content.

## 4. Materials and Methods

### 4.1. Peptides Synthesis and Purification

CPPs were synthesized with the use of an automated peptide synthesizer as C-terminal amides on TentaGel S RAM amide resin (0.25 mmol NH_2_/g capacity) using Fmoc chemistry and TBTU as a coupling reagent. Propynoic acid was coupled manually to the N-terminal end of the immobilized Tat peptide in the last step of the synthesis using a 10-fold molar excess of propiolic anhydride 30. The peptides were cleaved from the peptidyl-resin by treatment with a mixture of TFA/phenol/water/triisopropylsilane (TIPS) (88/5/5/2) at room temperature (RT) for 3 h under argon bubbles. The crude peptides were precipitated and washed with ice-cold diethyl ether. After lyophilization, the peptides were purified by preparative RP HPLC (Dr Maisch C-18 column with the dimensions of 250 mm × 40 mm, particles of 10 µm). Several gradient methods of acetonitrile (ACN) with 0.08% TFA with a flow rate of 25 mL/min were used throughout the purification. Fractions of the highest purity (>95%) were analyzed by analytical RP HPLC using a Phenomenex Kinetex XB-C18 column with the dimensions of 150 mm × 4.6 mm, particles of 5 µm with several gradient methods of ACN with 0.08% TFA and a flow rate of 1 mL/min. After purification and lyophilization, the peptides were dissolved in 0.01 M AcOH for trifluoroacetate to acetate ion exchange and relyophilized. Afterwards, the peptide homogeneity was characterized by analytical RP HPLC, capillary electrophoresis (CE), and ESI-MS using the QTOF 5600+ (Sciex, Framingham, MA, USA) mass spectrometer. The physicochemical properties of the peptides are summarized in Table 1.

### 4.2. Circular Dichroism Spectroscopy

Circular dichroism (CD) spectra of peptides were recorded on a Jasco J-815 spectropolarimeter (Jasco Int. Co., Ltd., Tokyo, Japan). The CD spectra were acquired for peptides in a concentration of 0.15 mg/mL in 10 mM PBS buffer (pH 7.0) or 30% (*v/v*) 2,2,2-trifluoroethanol (TFE) in 0.1 cm path length cuvettes and were measured in 185–250 nm spectral range at 25 °C. Ellipticity was measured in mdeg units. The spectra were averaged after three scans.

### 4.3. Molecular Dynamics Calculations

Peptide structures were constructed using standard modules from the AMBER 16 package [118]. Raw models were initially energy-minimized and submitted to the 100 ns unconstrained molecular dynamics (MD) simulation in the ff15ipq force field available in the AMBER suite of programs [119]. The MD simulations were conducted using two different solvents for each MD run: water and 30% (*v*/*v*) TFE aqueous solution. The standard pairwise generalized Born solvation model was used throughout all MD simulations. For the TFE solution, a dielectric constant of 43.06 was set [120]. Resulting trajectories were analyzed using the CPPTRAJ module of AMBER and hierarchical agglomerative (bottom-up) algorithm for clustering analysis [121]. Ten clusters were acquired for each trajectory. Representative structures of the two most populated clusters were obtained for each condition. All figures were evaluated using the MOLMOL 2K.2 software, date: 20 January 2003; [122].

### 4.4. Primary Neural Cortical Cultures

All animal experiments were conducted in accord with the permission of the Local Bioethics Committee. Primary cortical neurons were isolated from Wistar rat embryos at embryonic day 18.5 (E18.5). Pregnant females were euthanized in a CO_2_ chamber, and the embryos were immediately removed in order to microdissect the brains. The cortices were excised, transferred to Hank’s Balanced Salt Solution (HBSS, Gibco, Waltham, MA, USA), supplemented with a 1% penicillin/streptomycin mixture (Gibco, Waltham, MA, USA) and washed. Afterwards, the tissue was trypsinized (2.5% trypsin, Gibco, Waltham, MA, USA) for 10 min at 37 °C, and homogenized gently by re-suspension with a sterile tip of a pipette. The cell suspension was centrifuged in 300 rcf for 4 min and re-suspended in Neurobasal-A medium (Gibco, Waltham, MA, USA) supplemented to final concentration with 1 mM sodium pyruvate, 12.5 mM glucose 2% B27 (Gibco, Waltham, MA, USA), 200 mM L-glutamine, 10 mM glutamic acid, a 1% penicillin/streptomycin mixture, and seeded in a 24-well plate poly-D-lysine (PDL)-coated (BioCoat, Corning, Tewksburry, MA, USA) in the number of 200,000 cells per well. The neural cultures were incubated in a CO_2_ chamber (5%, 95% air balance, 98% humidity, 37 °C) for 14 days and were used on culture day in vitro (DIV) 14–15.

### 4.5. Optimization of an In Vitro Model of Acute Ischemic Stroke

#### 4.5.1. Optimization of Glucose Deprivation

For glucose deprivation, after removing culture medium from 24-well plates, neural cells were rapidly washed with 300 µL PBS (pH 7.4) and then incubated with 500 µL of glucose- and sodium pyruvate-free Neurobasal-A medium (described above) for 10 and 30 min, and 1, 2, and 3 h in a standard CO_2_ chamber. Each plate contained control cells that were incubated in a standard Neurobasal medium. Upon removal of the plates from an incubator, the media from all wells were aspirated and replaced with 500 µL of fresh, standard culture medium. The cells were cultivated in such conditions in a standard 5% CO_2_ chamber (37 °C) for 20 h.

#### 4.5.2. Optimization of the Sodium Azide-Induced Inhibition of Cellular Respiration

Oxidative stress was modeled by exposing neural cells to sodium azide to inhibit cellular respiration. After removing the culture medium from 24-well plates, the cells were rapidly washed with 300 µL of PBS and subsequently incubated with medium containing 1, 5, 10, 20, or 50 mM sodium azide for 1 h in a standard 5% CO_2_ chamber (37 °C). Afterwards, the medium was exchanged for 500 µL of standard Neurobasal medium, and the cells were incubated for 20 h in a 5% CO_2_ chamber (37 °C).

#### 4.5.3. Optimization of Acidosis

Culture medium for acidosis was optimized in terms of pH and concentration of sodium lactate. For optimization, culture media were aspirated from 24-wells and the cells were washed rapidly with 300 µL PBS (pH 7.4) and incubated with 500 µL of a Neurobasal of pH 7.4 (control), 6.5, 6.35, 5.55, 5.2, and 4.6. The pH was adjusted with 0.1 M HCl. The sodium lactate concentration was optimized similarly by incubating the cells in a medium containing 50, 200, and 1000 mM sodium lactate for 1 h. Finally, the synergistic effect of both factors was measured. In all cases, after 1 h, the stress-containing media were switched to fresh Neurobasal media and cells were incubated in a standard CO_2_ chamber (37 °C) for 20 h.

#### 4.5.4. Optimization of Excitotoxicity Models

Excitotoxicity was evaluated in three individual models, including glutamic acid, *N*-methyl-D-aspartate (NMDA) and kainic acid. All excitotoxic inducers were freshly prepared prior to the experiment and evaluated in concentrations of 400, 800, 1200, 1600, and 2000 µM. Application of the inducers followed the removal of the culture media from the 24-well plates and washing the cells with 300 µL PBS (pH 7.4). The cells were subsequently incubated for 1 h with a stressor in a concentration of 400, 800, 1200, 1600, and 2000 µM. Then, media were exchanged for 500 µL of a standard Neurobasal medium, and the cells were incubated for 20 h.

### 4.6. Treatment of Neural Cultures with Peptides

The influence of all peptides on neural viability was assessed by exposing the cells to different peptide concentrations (1, 10, 50, 100, 200 µM) for 20 h at 37 °C in a 5% CO_2_ chamber. The assessments were performed in 24-well PDL-coated plates (BioCoat, Corning, Tewksbury, MA, USA) with a working volume of 500 µL/well. The respective peptide solutions were prepared in culture medium so that in all wells only 50 µL of the medium was exchanged, including control (untreated) samples.

### 4.7. Pro-Viable Effect of the Peptides in an In Vitro Model of Acute Ischemic Stroke

The pro-viable properties of the tested peptides were studied in a neural model of brain injury, including glucose deprivation (Neurobasal-A, Gibco, Waltham, MA, USA), acidosis (pH 6.35, 200 mM sodium lactate), inhibition of cellular respiration (10 mM NaN_3_), and excitotoxicity (400 µM glutamic acid, 800 µM NMDA, 800 µM kainic acid; each analyzed independently). The activities of individual peptides were analyzed on two 24-well plates, each containing three 6-well rows for three insult conditions: one plate included excitotoxicity, the other included glucose deprivation, acidosis, and oxidative stress. The remaining row on each plate provided wells for control samples. On each plate, one half of each row was predestined to the treatment with the respective insult factor and then with the fresh medium. The other half of each row was treated with the same injury factor followed by incubation with the respective peptide. All insult factor media were freshly prepared before use, and cells were exposed to a 1 h incubation with an insult fact. Afterwards, the cells were carefully washed with 300 µL of PBS. The twelve wells that included the control and insult-treated samples were incubated with fresh culture medium, whereas the remaining wells were treated with a medium containing the respective peptides. Prior to use, the peptides were dissolved in Neurobasal medium in final concentrations of 50 µM. A 20 h incubation in standard conditions was followed with the neural viability assessment, as described -in the paragraph 4.8.

### 4.8. Neural Viability Assessments

Neural viability was assessed in CellTiter-Blue^®^ (CTB) assay (Promega, cat #G8080, Madison, WI, USA) after 20 h long incubation of cells in standard Neurobasal medium after stressful conditions. The test was performed in 24-well PDL-coated plates (BioCoat, Corning, Tewksbury, MA, USA) and according to the manufacturer’s guideline, which included adding the solution to the neural culture well, followed by incubation for 1–4 h at 37 °C (5% CO_2_). The fluorescence intensity of the CTB reagent was measured spectrofluorimetrically (560_ex_/590_em_) and was proportional to the number of viable cells. The results were calculated as a percentage taking the respective control (non-insulted) measurement as 100% viability.

### 4.9. Statistical Analysis

Each cell viability experiment was performed in at least three biologically independent experiments, in each of which neural cultures were obtained from one female rat. Each female originated from different breeding parents. In one experiment, the viability assessments for each condition were conducted in three plates, with three–four replicate wells per plate. For every plate, relative viability for one condition were averaged, and then used to calculate averaged results for all plates in an experiment. The final values were calculated from averaged results of independent experiments and shown as a mean ± standard deviation (SD), *n* = 3. The data were analyzed by a two-way ANOVA test with *p*-value < 0.05 values considered statistically significant.

## 5. Conclusions

**Figure 8:**
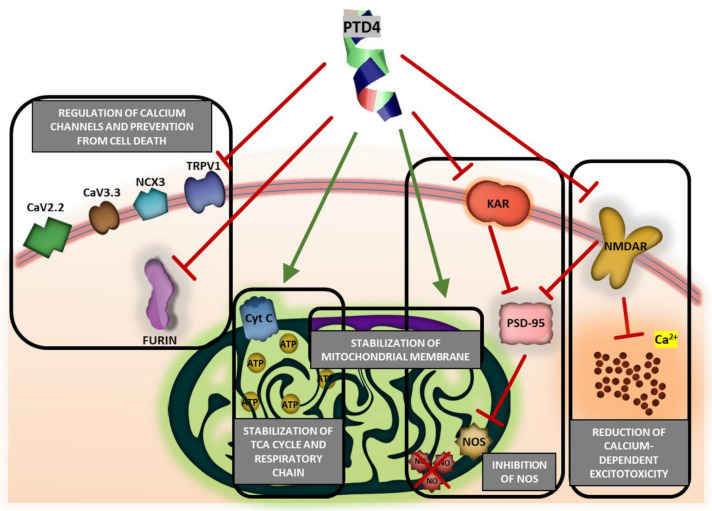
Conjectured mechanisms of PTD4-mediated neuroprotection based on the results of the study. PTD4–Y^47^ARAAARQARA^57^-amide; CaV2.2–N-type voltage-gated calcium channel; CaV3.3–T-type voltage-gated calcium channel; NCX3–sodium-calcium exchanger 3; TRPV1–transient receptor potential cation channel subfamily V member 1; Cyt C–cytochrome C; ATP–adenosine triphosphate; NOS–nitric oxide synthase; NO–nitric oxide; PSD-95–post-synaptic density protein 95; KAR–kainic acid receptor; NMDAR–N-methyl-D-aspartic acid receptor; TCA–tricarboxylic acid cycle.

Tat peptides containing the HIV-1 Tat arginine-rich motif demonstrated no neurotoxicity up to concentration of 50 µM. This outcome allows for the use of these cell-penetrating peptides as pro-viable and neuroprotective agents. Pro-viable properties of Tat(49–57)-NH_2_ and its analogue, PTD4, were verified in a multifactorial in vitro model of acute ischemic stroke. Significant effects of PTD4 against severe metabolic outcomes of AIS may suggest important mechanistic implications. Based on our results, we propose a conjectured mechanism of PTD4-mediated neuroprotection (Figure 8). Being less basic, PTD4 provided comparable yet broader neuroprotection as the native Tat(49–57)-NH_2_ peptide. This fact argues against a well-established mechanism relying on the crucial involvement of arginine guanidinium groups of RRPs’ interactions with the neuronal membrane. From this perspective, PTD4 seems to be the most suitable arginine-rich peptide in terms of neuroprotection among the tested peptides. In conclusion, PTD4 displayed mild pro-viable activity in ischemia-stricken neural cells. This propensity may predispose the PTD4 peptide to act as a leading targeting molecule attributed with inherent neuropharmacological activity against acute ischemic stroke.

## Figures and Tables

**Table 1:** Physicochemical properties of peptides studied (* asymmetrical dimethylation).

Peptide	Sequence	Remarks	Molecular Weight [g/mol]	Net Chargeat pH 7.0
Tat(49–57)-NH_2_	RKKR^52^RQRRR^57^-amide	Native sequence	1338.62	9
DR52	RKKrRQRRR-amide		1338.62	9
Tat6s	Ac-RRQRRR-amide	Ac-Tat(52–57)-NH_2_	968.13	5
Tat7s	Ac-R(Me)_2_RQRRR-amide (ASDM) *	Ac-[Arg(Me)_2_]Tat(52–57)-NH_2_	996.13	5
PTD4	YARAAARQARA-amide		1203.36	4
Prop-Tat	propiolyl-RKKRRQRRR^57^-amide	Prop-Tat(49–57)-NH_2_	1390.62	8
TP10	AGYLLGKINLKALAALAKKIL-amide		2181.75	5

## Data Availability

The datasets used and/or analyzed during the current study are available from the corresponding author on reasonable request.

## Supplementary Materials

The following are available online at https://www.mdpi.com/article/10.3390/ijms22116086/s1.

## Abbreviations

AIS	Acute ischemic stroke
ARM	Arginine rich motif
BBB	Blood–brain barrier
CD	Circular dichroism
CPP	Cell penetrating peptide
CTB	CellTiter-Blue
FDA	Food and drug administration
HAND	HIV-associated neurological disorder
HIV	Human immunodeficiency virus
MD	Molecular dynamics
NMDA	N-methyl D-aspartate
NMDAR	NMDA receptor
NMDAR-PSD	NMDAR postsynaptic density protein
NO	Nitric oxide
NOS	Nitric oxide synthase
OS	Oxidative stress
PTD	Protein transduction domain
PTM	Post-translational modification
RRP	Arginine rich peptide
rtPA	Recombinant tissue plasminogen activator
TAT	Transactivator of transcription
TFE	Trifluoroethanol
