# Supplementary material for: PTD4 Peptide Increases Neural Viability in an In Vitro Model of Acute Ischemic Stroke
Source: Int J Mol Sci. 2021 Jun 4;22(11):6086. doi: 10.3390/ijms22116086 (PMC8200211; doi:10.3390/ijms22116086)
Supplement: Supplementary file 1 [file ijms-22-06086-s001.zip › ijms-1224527-supplementary.pdf]

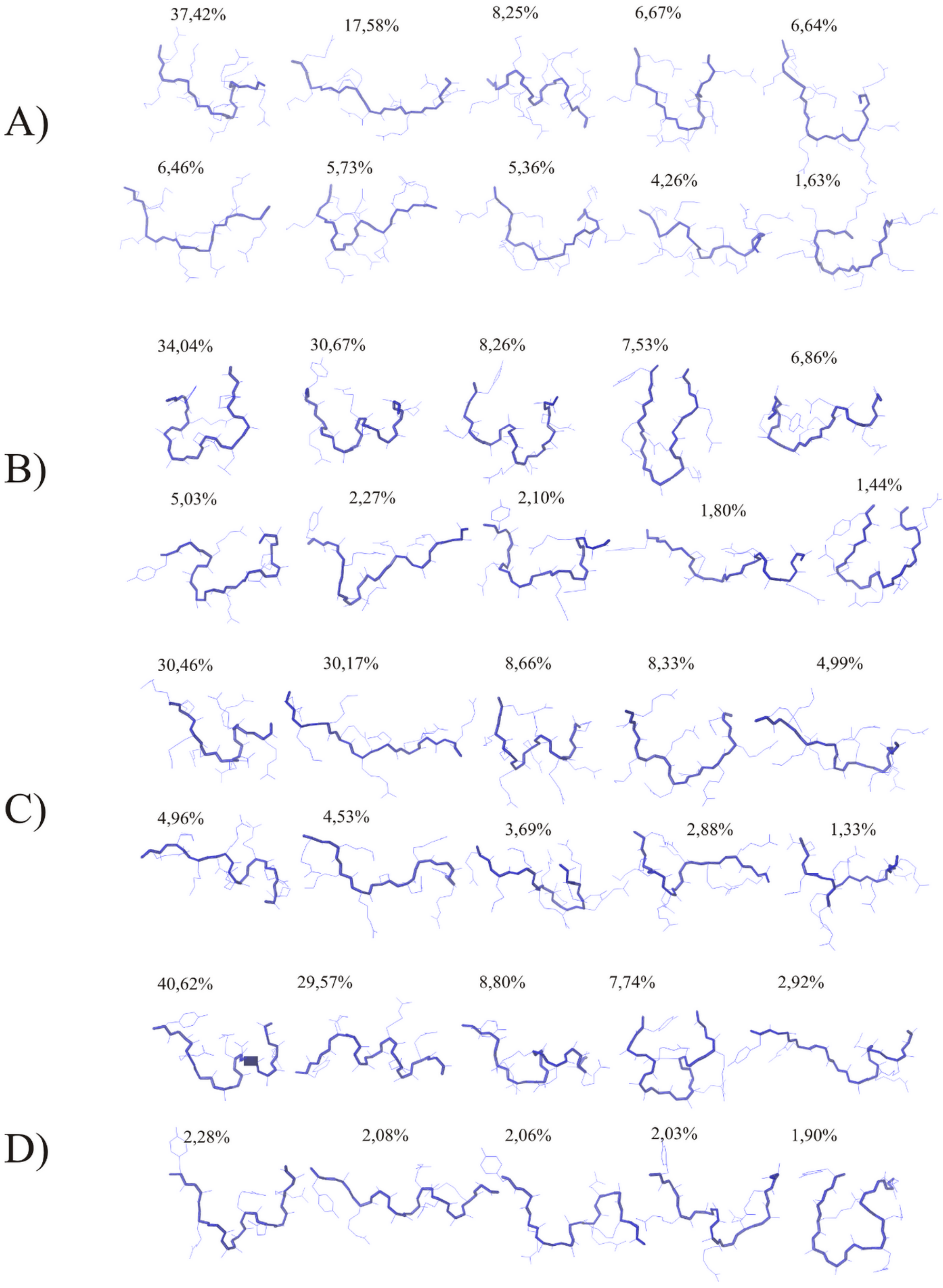

Figure S1. Ten clusters of 3D structures of **(A)** Tat(49-57)-NH_2 and **(B)** PTD4 peptides in water and of **(C)** Tat(49-57)-NH_2 and **(D)** PTD4 peptides 30 % (v/v) TFE, respectively. Each cluster is represented by one structure. All structural models were generated using the AMBER program.
